# Adsorption of Ternary Mixtures in the Presence of Multisite Occupancy: Theory and Monte Carlo Simulations

**DOI:** 10.3390/e27080849

**Published:** 2025-08-10

**Authors:** Pablo Jesús Longone, Antonio José Ramirez-Pastor

**Affiliations:** Departamento de Física, Instituto de Física Aplicada, Universidad Nacional de San Luis-CONICET, Ejército de Los Andes 950, San Luis D5700BWS, Argentina

**Keywords:** multisite-occupancy adsorption, lattice gas models, statistical thermodynamics, multicomponent gases

## Abstract

Adsorption of multicomponent mixtures on solid substrates is essential to numerous technological processes and provides key insights into surface phenomena. Despite advancements in theoretical modeling, many approaches still assume that each adsorbate occupies a single site, thereby neglecting important effects arising from molecules that span multiple adsorption sites. In this work, we broaden the theoretical description of such systems by considering the adsorption of *j* distinct polyatomic species on triangular lattices. Our approach is based on (i) exact thermodynamic results for polyatomic gases on one-dimensional lattices, extended here to account for substrates with higher coordination numbers, and (ii) the “0D cavity” functional theory originally developed by Lafuente and Cuesta, which reduces to the well-known Guggenheim–DiMarzio model in the limit of rigid rods. As a case study, we explore the behavior of a three-component system consisting of dimers, linear trimers, and triangular trimers adsorbing onto a triangular lattice. This model captures the interplay between structural simplicity, multisite occupancy, configurational diversity, and competition for space, key factors in many practical scenarios involving size-asymmetric molecules. We characterize the system using total and partial isotherms, energy of adsorption, and configurational entropy of the adsorbed phase. To ensure the reliability of our theoretical predictions, we perform Monte Carlo simulations, which show excellent agreement with the analytical approaches. Our findings demonstrate that even complex adsorption systems can be efficiently described using this generalized framework, offering new insights into multicomponent surface adsorption.

## 1. Introduction

Adsorption phenomena involving multicomponent mixtures on solid surfaces remain a central topic, not only due to their relevance in technological applications but also because of the fundamental insights they offer into surface interactions and structure formation [1,2,3,4]. While substantial progress has been made in the study of mixture adsorption, several important aspects remain unresolved. Experimental investigations of single-component adsorption are relatively straightforward and well-established, yet precise measurement techniques for gas mixtures often involve significant complexity and time investment [3,4,5,6].

From a theoretical perspective, a large proportion of existing models assume that each adsorbate species occupies a single adsorption site, thereby neglecting the possibility of multisite occupation in the adsorbed layer [5,6,7,8,9,10,11,12,13,14,15,16,17,18,19,20,21,22,23]. However, such simplifications can lead to inconsistencies when comparing theoretical predictions with experimental data. Crucial phenomena such as surface orientational phase transitions [24,25] or the displacement of one species by the other [26,27,28,29,30,31,32,33] cannot be adequately captured without including multisite occupancy effects. (This phenomenon, known as adsorption preference reversal (APR), is observed in systems such as methane–ethane mixtures [26,27]. APR involves a counterintuitive inversion in selectivity with pressure: ethane dominates adsorption at low pressure, whereas methane becomes predominant at higher pressures. Similar behavior has been reported for hydrocarbon mixtures in silicalite [28,29,30,31], carbon nanotube bundles [32], and MOFs [33]. APR arises due to the difference in molecular size and, consequently, site occupancy).

The lattice gas model [34] provides a valuable framework to address these limitations by accommodating extended adsorbates, such as linear molecules that cover multiple sites upon adsorption, often referred to as *k*-mers. Building on this foundation, a variety of methods have been employed to analyze systems with extended particle sizes. One of the earliest approaches is the Flory–Huggins (FH) approximation, independently formulated by Flory [35] and Huggins [36], which generalized the Bragg–Williams theory originally used for binary mixtures on two-dimensional lattices [34]. Designed to account for chain-like molecules, the FH model has undergone numerous refinements aimed at enhancing its accuracy, with comprehensive discussions provided in works such as [34,37]. Guggenheim proposed an alternative route for evaluating the partition function in these systems [38], which was later refined by DiMarzio through the inclusion of corrections for rigid rod-like molecules [39]. This extended treatment is now known as the Guggenheim–DiMarzio (GD) approximation.

In the 2000s, new theoretical formulations have emerged to better describe adsorption with multisite occupancy. The first, termed the Extension Ansatz (EA) approximation, uses analytically derived expressions for the thermodynamic properties of *k*-mers adsorbed on one-dimensional lattices and extends these results to more complex geometries [40,41]. A second approach, the Fractional Statistical Theory of Adsorption (FSTA) [42], is inspired by Haldane’s generalized exclusion statistics [43,44]. The third, the Occupation Balance (OB) method, employs a fugacity expansion to approximate adsorption thermodynamics [41,45]. Finally, the Semi-Empirical (SE) scheme combines exact 1D solutions with the GD framework to provide practical yet accurate predictions [46,47].

More recently, the Multiple Exclusion (ME) statistics framework was introduced to describe classical systems in which particles access spatially correlated states [48,49,50]. ME statistics accounts for situations in which multiple particles simultaneously exclude access to a common state, an intrinsic feature of non-monomeric particles on a lattice.

Despite the advances outlined above, most studies have concentrated on single-species adsorption, leaving the adsorption of *k*-mer mixtures relatively underexplored. Addressing this gap, our group has recently contributed a series of theoretical studies focused on polyatomic binary mixtures [51,52,53]. The first of these works [51] presents an exact statistical thermodynamic treatment for mixtures composed of *s*-mers and *k*-mers adsorbed on one-dimensional homogeneous substrates. This formalism, developed specifically for zeolite-like systems, was the first to rigorously model the adsorption of polyatomic mixtures and provided a clear theoretical explanation for the APR phenomenon. The analysis demonstrated that size asymmetry between adsorbed species plays a decisive role in driving APR and highlighted the risk of misinterpreting experimental data when the polyatomic nature of adsorbates is ignored.

A subsequent work [52] extended the analysis to two-dimensional lattices, using a generalized lattice gas approach informed by the classical GD approximation [38,39]. This framework enabled the exact solution in one dimension and yielded accurate approximations for higher dimensions, preserving the key effects of multisite occupation. In a third contribution [53], multicomponent adsorption of polyatomics was reframed within the language of fractional statistics, applying Haldane’s formulation [43,44] to model monomer–dimer mixtures and offering quantitative insight into methane–ethane adsorption in nanoporous materials such as zeolites.

In 2002, Lafuente and Cuesta reformulated Rosenfeld’s Fundamental Measure Theory (FMT) for application to lattice systems, starting from an exact density functional derived for one-dimensional mixtures of hard rods [54,55]. This novel formulation, known as Lattice Fundamental Measure Theory (LFMT), proved effective in generating highly accurate phase diagrams for mixtures of hard-core particles [54,55,56]. Furthermore, the framework allows for the use of dimensional crossover techniques. This advantage was illustrated through the derivation of density functionals for lattice gases with nearest-neighbor exclusion, applicable across various lattice geometries such as square, triangular, and both face- and body-centered cubic lattices, by projecting from a functional defined for hypercubic particles in (d + 1) dimensions [57]. The generality observed in these results led to a broader strategy for formulating FMTs suitable for arbitrary lattice structures, particle shapes, and spatial dimensionalities [58,59].

The results presented in Refs. [51,52,53] are restricted to two-component systems. In this work, we extend the theoretical framework of multicomponent adsorption by analyzing the case of *j* distinct species adsorbing on triangular lattices. To this end, we employ the EA approximation as our core methodological approach [41,47]. This method begins with the exact calculation of the partition function for a multicomponent gas of polyatomic species adsorbed on a one-dimensional lattice [40,51]. Building on this foundation, we incorporate established theoretical arguments [34,35,37,40,60,61] to generalize the configurational entropy to systems with higher lattice connectivity. The key correction factor introduced in this generalization quantifies the number of possible configurations per site for placing a *k*-mer at zero coverage and explicitly depends on both the lattice topology and the geometry of the adsorbing species.

In addition to the EA approximation, our results will also be discussed in the context of Lafuente and Cuesta’s theory [54,55,56,57,58,59]. As discussed in Ref. [62], for the case of straight rigid rods, the theory by Lafuente and Cuesta in the “0D cavities” approximation coincides with Guggenheim–DiMarzio (GD) theory [38,39]. This combined approach (referred to in this paper as GD-LC) will be applied to compute adsorption isotherms and the configurational entropy of the adsorbed phase.

**Figure 1:**
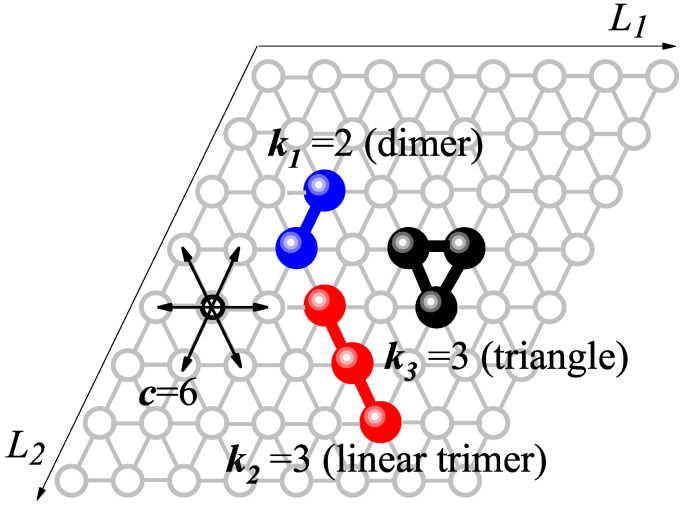
Schematic diagram showing the different adsorbate configurations studied in this work: dimer ($k_{1}=2$, blue symbols); linear trimer ($k_{2}=3$, red symbols); and triangular trimer ($k_{3}=3$, black symbols). Spheres and open circles represent $k_{i}$-mer’s units and empty sites, respectively.

With the general theoretical framework in place, we focus on a specific case study: the adsorption of a ternary mixture consisting of dimers, linear trimers, and triangular trimers on triangular lattices (see molecular structures in Figure 1). Three main motivations drive this choice. First, (i) the dimer represents the simplest polyatomic adsorbate and captures the essential features of multisite occupancy; (ii) the trimer is the simplest species exhibiting multiple adsorption configurations on triangular lattices; and (iii) the difference in molecular size between dimers and trimers leads to competitive displacement effects, a hallmark of multicomponent adsorption phenomena.

Second, recent work by our group [63,64,65] introduced triangular lattice models to study the stability and distortion of sI clathrate hydrate structures formed by methane and carbon dioxide. In these models, methane is represented as triangular trimers and carbon dioxide as linear trimers, revealing lattice deformation mechanisms and free energy landscapes. Although prior investigations focused on single-component systems, practical scenarios often involve coadsorption. One of the key open questions is how carbon dioxide displaces methane during clathrate formation, a process of both theoretical and practical relevance.

Third, and equally important, triangular lattices are common in both natural and engineered materials. Therefore, adsorption studies on such geometries have significant theoretical and experimental implications, particularly for understanding surface phase transitions and for the structural characterization of solid surfaces [66,67,68,69,70,71].

Our approach combines analytical modeling with Monte Carlo (MC) simulations to test the theoretical predictions. This combined methodology enhances the robustness of our conclusions by allowing direct comparison between analytical results and computational data.

The present study represents a natural progression from our earlier work [51,52,53], which focused on simple multicomponent systems, including binary mixtures and linear molecules. As a precursor, the adsorption behavior of pure gases composed of dimers, linear trimers, and triangular trimers was analyzed in Ref. [72]. The problem becomes substantially more complex, and scientifically richer, when these three species are allowed to interact and adsorb concurrently.

The remainder of this paper is structured as follows: The model and the Monte Carlo simulation methodology are detailed in Section 2. In Section 3, we present and discuss the results. Section 4 summarizes our main conclusions. Appendix A presents the thermodynamic framework for multicomponent lattice gases with hard-core interactions. Finally, details of the Ideal Adsorbed Solution Theory (IAST) are given in Appendix B.

## 2. Model and Monte Carlo Simulation Scheme

A monolayer adsorption scenario is examined, involving a *j*-component mixture, where the system contains $N_{1}$ molecules of type 1, $N_{2}$ of type 2, …, and $N_{j}$ of type *j*. Each molecule of species *i* is a linear $k_{i}$-mer, meaning it is composed of $k_{i}$ identical segments, each occupying one lattice site; thus, a $k_{i}$-mer covers exactly $k_{i}$ consecutive lattice sites upon adsorption. Interactions are limited strictly to steric hindrance: no overlap between $k_{i}$-mers is permitted, ensuring that no site is shared by multiple adsorbed units.

Systems governed exclusively by steric constraints hold particular significance in statistical mechanics because their potential energy, *U*, remains constant by definition. As a result, the Helmholtz free energy, F=U−TS, becomes entirely dependent on the entropy, *S*, meaning that any phase behavior arises solely from entropic effects. In a seminal contribution, Onsager [73] demonstrated that systems composed of extremely elongated rods with purely excluded volume interactions can undergo entropy-driven transitions to nematic phases exhibiting long-range orientational order. The problem proposed by Onsager is a clear example of an entropy-driven phase transition. Other examples of entropy-driven systems can be found in Refs. [74,75,76]. In closer connection to the present study, recent investigations on antifreeze proteins [77] and guest exchange in clathrate hydrates [65] have shown that complex adsorption and displacement phenomena can emerge purely from steric and entropic mechanisms. These systems exhibit competition and selectivity without the need for explicit energetic interactions. This perspective reinforces the relevance of entropic models based solely on geometric constraints and multisite occupancy, such as the one we use here.

In order to model the adsorption process of the mixture, a general Monte Carlo (MC) scheme in the grand canonical ensemble was implemented [78,79]. The surface was represented by a triangular lattice of M=L×L adsorption sites, and a ternary (three-component) mixture was selected as the subject of study. The mixture consists of dimers (species 1, $k_{1}=2$), linear trimers (species 2, $k_{2}=3$), and triangular-shaped trimers (species 3, $k_{3}=3$). These species are adsorbed along three lattice directions and six possible orientations (connectivity c=6). Figure 1 provides a schematic representation of species 1 through 3. Blue spheres denote dimers, red spheres represent linear trimers, and black spheres correspond to triangular trimers. Unoccupied lattice sites are indicated by empty circles. The molar composition *X* of the ternary mixture satisfies the relation $X_{\mathrm{Total}}=X_{1}+X_{2}+X_{3}$= 1.

As mentioned at the beginning of this section, the only interaction between different adsorbed particles is hard-core exclusion. In addition, since the lattice is assumed homogeneous, $E(M,N_{1},N_{2},N_{3})$ (Equation (A5)) can be arbitrarily chosen equal to zero without losing generality (i.e., the interaction energy between every $k_{i}$-mer and the substrate is set to be zero, $\epsilon_{i}=0$ for i=1,2,3).

Then, given a lattice of *M* equivalent adsorption sites in contact with a ternary gas mixture at temperature *T* and pressure *P*, the state of the system may change through the adsorption or desorption of a $k_{i}$-uple ($k_{i}$-mer) of any of the three species. An elementary MC simulation step (MCS) proceeds as follows:

1. **Initialization:**
  - Set temperature *T*, pressure *P*, and molar compositions $X_{1}$, $X_{2}$, and $X_{3}$. Accordingly,
    (1) $$\mu_{i}=\mu_{i}^{0}+\ln X_{i}P\;\;\;\;\left[i=1\;(\mathrm{dimer}),\;i=2\;(\mathrm{linear}\;\mathrm{trimer})\;\mathrm{and}\;i=3\;(\mathrm{triangular}\;\mathrm{trimer})\right].$$
2. **Random species selection:**
  - Randomly select one of the three species.
3. **Random $k_{i}$-uple selection:**
  - Randomly select a set of $k_{i}$ sites on the lattice forming a $k_{i}$-uple, according to the geometry of the species selected in step 2.
    - If the $k_{i}$-uple is empty, attempt to adsorb a $k_{i}$-mer of the type selected in step 2 with probability $W_{\mathrm{ads}}$.
    - If the $k_{i}$-uple is fully occupied by a $k_{i}$-mer of the type selected in step 2, attempt to desorb it with probability $W_{\mathrm{des}}$.
    - If the $k_{i}$-uple is partially occupied, or fully occupied, by elements belonging to different adsorbed particles, the attempt is rejected.
  - The probabilities $W_{\mathrm{ads}}$ and $W_{\mathrm{des}}$ are calculated according to the Metropolis criterion [78,79]:
    (2) $$W_{\mathrm{ads}}=\min\left[1,P\exp\left(-\frac{\Delta E}{k_{B}T}\right)\right],$$
    and
    (3) $$W_{\mathrm{des}}=\min\left[1,\frac{1}{P}\exp\left(-\frac{\Delta E}{k_{B}T}\right)\right],$$
    where ΔE is the difference between the energies of the final (new) and initial (old) states.
4. **Repeat the simulation step:**
  - Repeat steps 2–3 a total of *M* times to complete one MCS.

In our MC simulations, the equilibrium state can be well reproduced after discarding the first $m^{\prime }=10^{6}$ MCS. Then, averages are taken over $m=10^{6}$ MCS successive configurations. The initial configuration of the system is an empty triangular lattice, and the final configuration obtained for a given pressure is used as the initial configuration for the next (higher) pressure.

Partial and total adsorption isotherms are obtained as simple averages

(4) $$\theta_{i}=\frac{k_{i}\langle N_{i}\rangle }{M},$$

and

(5) $$\theta =\sum_{i=1}^{i=j}\theta_{i},$$

where 〈…〉 means the average over the MC simulation runs.

The use of MC techniques for computing thermal averages of thermodynamic quantities is a well-established and powerful approach [78,79]. Observables such as total energy, energy fluctuations, and correlation functions can be reliably estimated by averaging over a sufficiently large set of sampled configurations. In contrast, quantities like the free energy and entropy are more elusive, as they are not directly accessible through simple ensemble averages. To overcome this challenge, several indirect approaches have been introduced [79], with thermodynamic integration being among the most effective and commonly applied methods [45,80,81,82].

Within the grand canonical framework, thermodynamic integration involves evaluating the chemical potential μ as a function of surface coverage, tracing a reversible path from a known reference state to the target state of interest. This procedure also requires prior knowledge of the Helmholtz free energy $F_{0}$ associated with the reference condition. Accordingly, for a system consisting of a single component with *N* particles distributed over *M* lattice positions, the free energy can be determined from the following expression:

(6) $$\mu ={\left(\frac{\partial F}{\partial N}\right)}_{M,T},$$

and

(7) $$F\left(M,N,T\right)=F_{0}\left(M,N_{0},T\right)+\int_{N_{0}}^{N}\mu dN.$$

Using F=E−TS, the configurational entropy *S* can be written as

(8) $$S\left(M,N,T\right)=S_{0}\left(M,N_{0},T\right)+\frac{E\left(M,N,T\right)-E\left(M,N_{0},T\right)}{T}-\frac{1}{T}\int_{N_{0}}^{N}\mu dN.$$

In the case of a *j*-component mixture containing $N_{1}$ molecules of component 1, $N_{2}$ molecules of component 2, …, $N_{j}$ molecules of component *j*, Equation (7) must be rewritten in terms of the chemical potentials of each adsorbed species $\mu_{i}$,

(9) $$\mu_{i}={\left(\frac{\partial F}{\partial N_{i}}\right)}_{N_{j},T,M}.$$

Integrating the last equation, we obtain

(10) $$F\left(M,N_{1},N_{2},\ldots ,N_{j},T\right)=F_{0}\left(M,N_{10},N_{20},\ldots ,N_{j0},T\right)+\sum_{i=1}^{j}\left(\int_{N_{i0}}^{N_{i}}\mu_{i}dN_{i}\right).$$

Accordingly, it follows that

(11) $$\begin{array}{ccc} S(M,N_{1},N_{2},\ldots ,N_{j},T) & = & S_{0}\left(M,N_{10},N_{20},\ldots ,N_{j0},T\right)\\ & & +\frac{E\left(M,N_{1},N_{2},\ldots ,N_{j},T\right)-E\left(M,N_{10},N_{20},\ldots ,N_{j0},T\right)}{T}\\ & & -\frac{1}{T}\left[\sum_{i=1}^{j}\left(\int_{N_{i0}}^{N_{i}}\mu_{i}dN_{i}\right)\right]. \end{array}$$

In our case $E(M,N_{1},N_{2},\ldots ,N_{j},T)=0$ and the determination of the entropy in the reference state, $S_{0}\left(M,N_{10},N_{20},\ldots ,N_{j0},T\right)$, is trivial [$S_{0}\left(M,N_{10},N_{20},\ldots ,N_{j0},T\right)=0$ for $N_{10}=N_{20}=\cdots =N_{j0}=0$]. Then,

(12) $$S\left(M,N_{1},N_{2},\ldots ,N_{j},T\right)=-\frac{1}{T}\left[\sum_{i=1}^{j}\left(\int_{0}^{N_{i}}\mu_{i}dN_{i}\right)\right].$$

After writing the last equation in terms of intensive variables, the configurational entropy per site (s=S/M) results in

(13) $$\frac{s(\theta ,T)}{k_{B}}=-\frac{1}{k_{B}T}\left[\sum_{i=1}^{j}\left(\int_{0}^{\theta_{i}}\frac{\mu_{i}}{k_{i}}d\theta_{i}\right)\right],$$

where $\theta_{i}=k_{i}N/M$ and $\theta =\sum_{i}\theta_{i}$.

The $\mu_{i}$ versus $\theta_{i}$ curves are determined by applying the adsorption–desorption methodology outlined earlier in this section. The integration involved in Equation (13) is performed using the trapezoidal approximation, a standard numerical technique [83]. Since all chemical potentials are expressed in units of $k_{B}T$, all results will be independent of the temperature. Therefore, throughout the remainder of this work, we will refer to s(θ) as the configurational entropy per lattice site, omitting the explicit temperature reference (for simplicity we will drop the “*T*”).

The simulation scheme developed in this section was tested on one-dimensional lattices, where the model presented in Appendix A yields exact results. Indistinguishable outcomes were obtained from both theoretical calculations and Monte Carlo (MC) simulations. This agreement validates the MC simulation framework, demonstrating that the kinetic rules outlined in steps (1–4) (Equations (1)–(3)) produce equilibrium states that are in direct correspondence with predictions from statistical mechanics. However, these results cannot be directly related to real-time dynamics. To accurately simulate the time evolution of real physical processes, it is necessary to employ the Kinetic Monte Carlo method [79]. In this context, future work will focus on developing an *n*-fold way Monte Carlo algorithm [84], enabling the investigation of the key kinetic properties of these multicomponent systems under conditions of multiple site occupancy.

## 3. Results

In this section, the adsorption isotherm (i.e., surface coverage as a function of pressure) and the configurational entropy per site of the adsorbed layer, as predicted by the EA and GD-LC theoretical models, are compared with the results obtained from MC simulations, following the procedure outlined in Section 2.

Within the framework of the EA theoretical approach, it is important to emphasize that the use of the approximate configurational factor $\Omega_{c}\left(M,N_{1},N_{2},\ldots ,N_{j}\right)$ (Equation (A4)) enables a direct evaluation of the Helmholtz free energy. As a consequence, the partial and total thermodynamic functions of the ternary mixture, corresponding to different gas-phase compositions, can be consistently derived via Equation (A17).

On the other hand, the numerical evaluation of the entropy per site, $s/k_{B}$, using Equation (13), is straightforward and computationally efficient, since the coverage dependence on $\mu /k_{B}T$ is computed according to the MC simulation protocol described in Section 2. In this approach, the partial chemical potentials are integrated as a function of the partial coverage of each species.

The simulations were carried out on triangular lattices of size L×L, with L=360. To minimize boundary effects, periodic boundary conditions were applied. Under these conditions, finite-size effects—which could influence adsorption isotherms in smaller systems—were found to be negligible. These findings are consistent with the results reported in a previous study by our research group [72], where a type-cluster approximation [85,86,87,88] was combined with MC simulations to analyze the configurational entropy per site of dimers and trimers on triangular lattices. It was observed that surface coverage values remain practically unchanged for L>90.

This section is organized into two main parts. In the first part (Section 3.1), a comprehensive comparison is made between the adsorption isotherms and configurational entropy per site obtained from the EA theoretical model (Appendix A), the GD-LC theory, the Ideal Adsorbed Solution Theory (IAST) [5,6], and the MC simulations (Section 2). Two representative cases are examined for (dimers–linear trimers–triangular trimers) ternary mixtures: an equimolar composition ($X_{1}=X_{2}=X_{3}=1/3$) and a diluted case for dimers ($X_{1}=0.10$) with equal fractions of linear and triangular trimers ($X_{2}=X_{3}=0.45$). This section also includes the study of the behavior of the partial configurational entropy per site, with particular emphasis on the role of this quantity in the displacement of larger species (trimers) due to the presence of smaller ones, specifically, dimers. The studied cases provide insights into the evolution of the partial entropy of each species and allow for a better understanding of their individual and collective contributions to the displacement process.

In the second part (Section 3.2), the total entropy of the mixture is examined as a function of different molar fractions of dimers (k=2). This analysis sheds light on how the total entropy landscape varies with composition, offering a broader perspective on the thermodynamic response of the system.

In all three parts of this study, the adsorbed species are treated as ideal, meaning that no lateral interactions between adsorbates are considered. The only interaction accounted for is excluded volume, which arises from the differences in size and shape among the species adsorbed on the lattice.

### 3.1. Adsorption Isotherms and Total Configurational Entropy per Site

We begin by analyzing a ternary mixture system by comparing results from the MC simulations described in Section 2 with predictions from EA approximation, the GD-LC theoretical model, and the well-established Ideal Adsorbed Solution Theory (IAST) for ideal mixtures [5,6].

**Figure 2:**
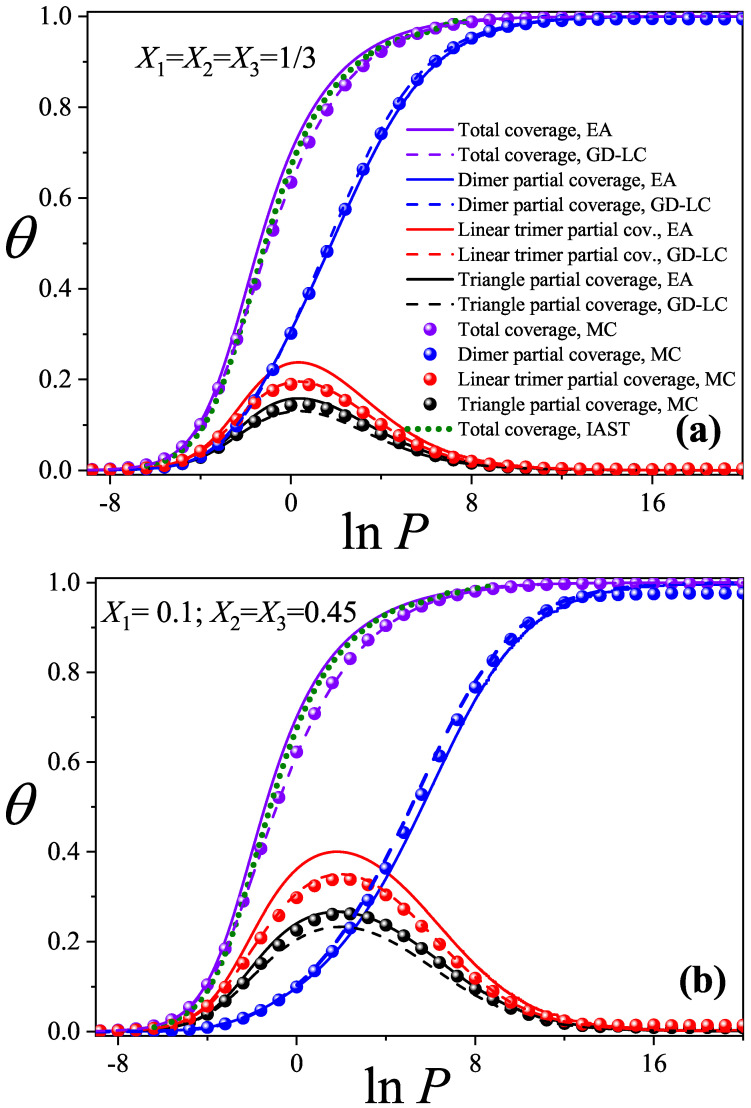
Comparison of partial and total adsorption isotherms obtained from the EA approximation, GD-LC theoretical model, MC simulations, and IAST calculations for ideal ternary mixtures with two different sets of molar compositions $X_{i}$. (**a**) $X_{1}=X_{2}=X_{3}=1/3$; (**b**) $X_{1}=0.10$, $X_{2}=X_{3}=0.45$. The symbols used are explained in the inset of part (**a**).

Figure 2a,b display the total and partial adsorption isotherms for two different molar composition sets: (a) $X_{1}=X_{2}=X_{3}=1/3$ and (b) $X_{1}=0.10$, $X_{2}=X_{3}=0.45$. Solid lines correspond to theoretical predictions obtained from Equation (A18) (EA approximation); green dashed lines represent IAST results; and sphere-shaped markers indicate data from MC simulations. Curves for dimers are shown in blue, linear trimers in red, and triangular trimers in black.

The GD-LC results, depicted as dashed lines, are obtained by applying the Guggenheim–DiMarzio (GD) scheme [38,39] to the ternary mixture under study. As mentioned in Section 1, the GD and LC approaches (within the “0D” cavity framework) coincide in the case of straight rigid rod adsorbates. Under these conditions, the expressions derived for the partial isotherms resemble Equation (A18), with a single modification: the term $\left(k_{i}-1\right)\ln\left[1-\sum_{l=1}^{j}\left(\frac{k_{l}-1}{k_{l}}\right)\theta_{l}\right]$ is replaced by $\left(k_{i}-1\right)\ln\left[\frac{c}{2}-\sum_{l=1}^{j}\left(\frac{k_{l}-1}{k_{l}}\right)\theta_{l}\right]$, where c=6 for triangular lattices [52].

**Figure 3:**
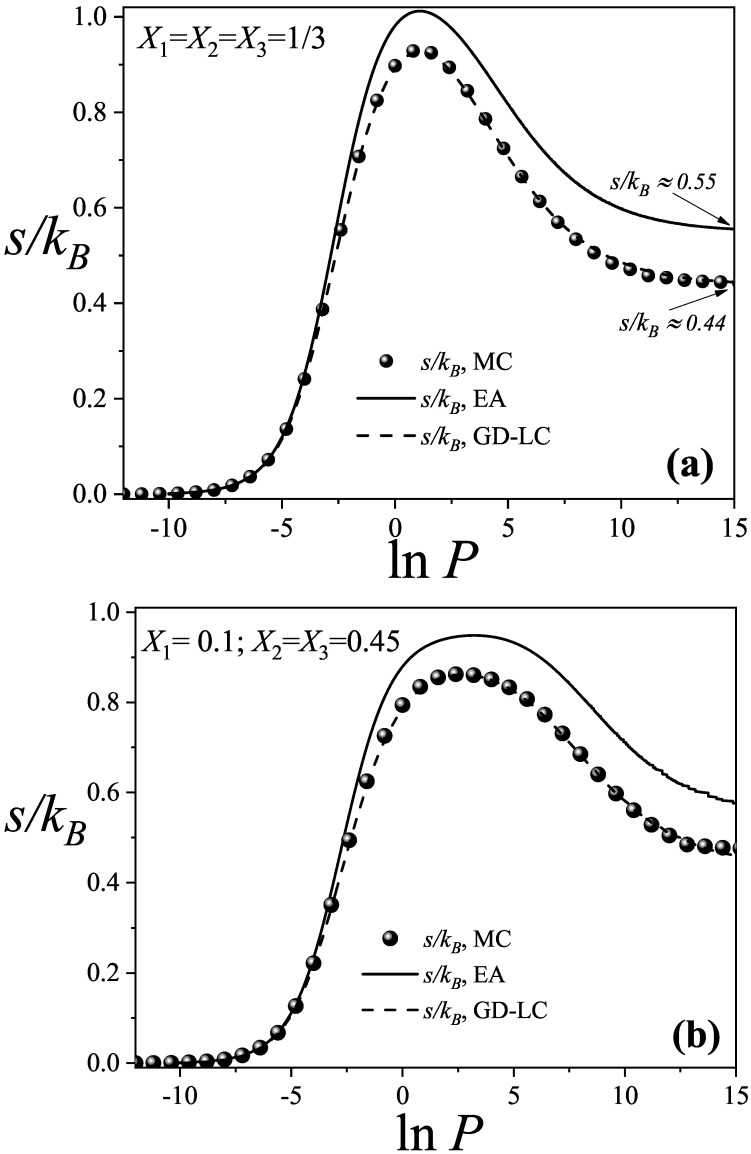
Configurational entropy per site as a function of pressure (in ln scale) from the EA approximation (solid lines), GD-LC theoretical model (dashed lines), and MC simulations (spheres) for the same cases studied in Figure 2. Symbols are defined in the inset.

In addition, for triangular trimers, a correction factor is introduced to account for their nonlinear geometry. This factor is obtained by following reasoning similar to that used in Equation (A4). Namely, while a linear trimer has c/2 accessible states per site at zero coverage (corresponding to the three directions of the triangular lattice), a triangular trimer has only two accessible states per site (with the triangle pointing either upwards or downwards). It then follows directly that the correction factor to be introduced into the partial isotherm of the triangular trimer is ln(c/2)−ln2. As we will show in Figure 2 and Figure 3, this theoretical scheme yields excellent agreement with MC results.

For both molar composition sets $X_{i}$, the theoretical models exhibit excellent qualitative agreement with the Monte Carlo (MC) simulations for both total and partial adsorption isotherms. In particular, the GD-LC model exhibits outstanding quantitative agreement with MC results. Only very slight deviations are observed in the partial isotherms of the triangular trimer species, while no such discrepancies appear for the dimer or linear trimer species.

Additionally, the total adsorption isotherms calculated using IAST show closer agreement with those of the theoretical model than with those of MC simulations. This behavior likely stems from the fact that IAST is constructed on ideal pure-component isotherms based on the configurational factors developed in Equation (A4). For further details on the IAST approach, see Appendix B.

Moreover, for both cases studied (equimolar and asymmetric mixtures), a clear displacement of larger species (trimers) by smaller ones (dimers) is observed. As lnP increases, species with $k_{i}=2$ dominate the adsorption process on the triangular lattice. In the high-pressure limit (large lnP), the total coverage θ is almost entirely due to dimer adsorption, with both linear and triangular trimers being nearly absent. This displacement effect is accurately captured by both the EA and GD-LC theoretical approaches.

Figure 3 shows the total configurational entropy per site for the same systems analyzed in Figure 2. Solid lines represent theoretical predictions, while spheres correspond to MC results. In the theoretical model, the configurational entropy per site is calculated using Equation (A14), with $\theta_{i}$ determined from Equation (A15). In the GD-LC and MC approaches, the total entropy of the mixture is obtained as the sum of the partial entropies of each species, given by

(14) $$\frac{s(\theta_{1},\theta_{2},\ldots ,\theta_{j},T)}{k_{B}}=\sum_{i=1}^{j}\frac{s_{i}\left(\theta_{i},T\right)}{k_{B}},$$

where j=3 in this case, and each $\frac{s_{i}\left(\theta_{i},T\right)}{k_{B}}$ is computed by integrating the partial chemical potential $\mu_{i}$ as a function of the partial coverage $\theta_{i}$ for species *i*.

Figure 3a,b show excellent qualitative agreement between the theoretical models and MC simulations across both molar composition sets $X_{i}$. Notably, the GD-LC theory demonstrates superior performance, with its curves being nearly indistinguishable from the simulation results across the entire pressure range. In particular, the GD-LC model provides highly accurate entropy predictions in the high-pressure regime.

In contrast, the EA approximation exhibits a clear quantitative discrepancy when compared to the MC simulation data. The primary source of this discrepancy lies in the overestimation of accessible states for linear trimer species. As the occupancy of linear sites increases, the EA theoretical model tends to overcount configurations, particularly for larger species. This overcounting results from the breakdown of the approximations in $\Omega_{c}\left(M,N_{1},N_{2},\ldots ,N_{j}\right)$, which fail to fully capture the geometric constraints that emerge with increasing molecular size.

Despite the limitations discussed above, the theoretical predictions are considered highly satisfactory, especially given the significant complexity of modeling a ternary system composed of species with different sizes and multiple site occupancies.

The high-pressure regime is especially noteworthy, as the configurational entropy reaches a saturation value that becomes constant in both cases studied. Within the EA theoretical framework, this saturation value approaches $s/k_{B}\approx 0.5500$ for both compositions. In contrast, the GD-LC theory predicts a saturation entropy of $s/k_{B}\approx 0.4390$, which is remarkably close to the value obtained from MC simulations, $s/k_{B}\approx 0.4428$. This latter result is especially significant, as it closely matches the value reported by other authors for a triangular lattice fully occupied by dimers, $s/k_{B}=0.428594537\ldots$ [89]. This close agreement provides further evidence that, in the high-pressure regime, the lattice is effectively saturated by dimers.

For a lattice saturated with linear trimers, the configurational entropy per site was reported as $s/k_{B}=0.201\left(4\right)$ [72], based on Monte Carlo simulations combined with thermodynamic integration method. On the other hand, the exact entropy value for a lattice fully occupied by triangular trimers was calculated by Verberkmoes and Nienhuis as $s/k_{B}=\frac{1}{3}\ln\left(\frac{3}{4}\sqrt{3}\right)\approx 0.08720802396$ [90,91]. These findings not only reinforce the conclusion that the fully packed state corresponds predominantly to dimer occupation but also demonstrate that maximizing entropy at high surface coverage, or equivalently, minimizing the free energy, favors configurations where dimers are the dominant species, as opposed to those primarily composed of linear or triangular trimers.

The displacement of larger species (trimers) by smaller ones (dimers) has also been observed in studies of three-domain antifreeze proteins adsorbed on surfaces, where the molecules can bind via one, two, or all three domains—referred to as S1, S2, and S3 states, respectively [77]. The displacement of molecules in S2 and S3 by those in S1 is known as the APR phenomenon, previously reported in Refs. [26,27]. It is important to note that the partial coverage of the larger species tends toward zero but does not vanish entirely. In fact, a small residual adsorption of trimer species remains, on the order of $\theta \approx 1\times 10^{-3}$. This minor contribution accounts for the observed discrepancy between our MC saturation entropy and the value reported in the literature, resulting in a deviation of Δs=0.0142.

To further investigate the behavior of the total configurational entropy of the mixture, it is useful to compare Figure 2 and Figure 3. As can be observed from the figures, the partial adsorption isotherms of the larger species ($k_{i}=3$), namely, linear and triangular trimers, exhibit a maximum in coverage that coincides with the peak of the mixing entropy within the same lnP interval. In this region, the coverage of the smallest species (dimers, $k_{i}=2$) begins to increase with lnP, indicating the onset of a displacement process. Beyond the entropy maximum, the smaller species progressively displace the larger ones, gaining occupancy on the lattice.

This maximum in the mixing entropy corresponds to a relative minimum in the Helmholtz free energy, establishing a favorable thermodynamic condition under constant volume *M* and temperature *T*. From this point onward, the adsorption of the smaller species proceeds in a spontaneous, natural, and thermodynamically viable manner.

**Figure 4:**
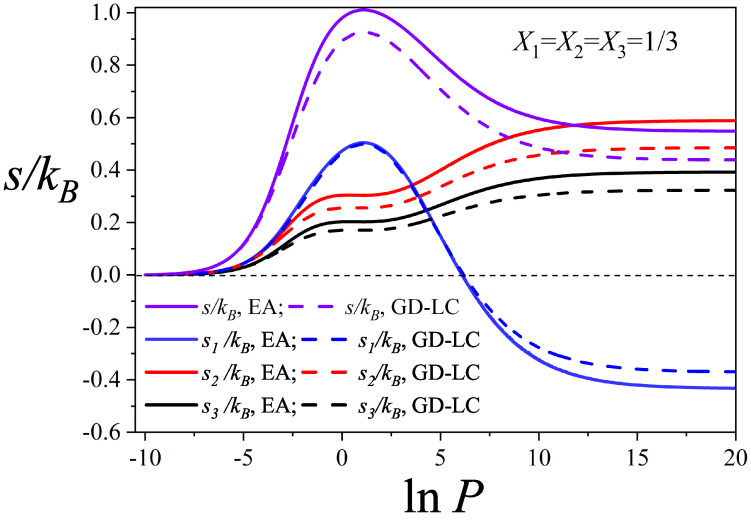
Total and partial configurational entropy per site as a function of lnP for the equimolar mixture studied in Figure 2a and Figure 3a: $X_{1}=X_{2}=X_{3}=1/3$. The symbols used are explained in the inset.

In Figure 4, the behavior of the partial configurational entropies as a function of lnP is shown, along with the total configurational entropy of an equimolar ternary mixture ($X_{1}=X_{2}=X_{3}=1/3$). This figure illustrates how the overall entropy curve of the mixture emerges from the combined contributions of each species across the entire pressure range. The curves were computed using the EA (solid lines) and GD-LC (dashed lines) theoretical models. Both models exhibit qualitatively similar behavior (in terms of curve shapes, locations of the maxima, and relative differences); however, quantitative discrepancies are observed, with the GD-LC model being the more accurate one, as discussed in Figure 3. The description that follows applies to both models.

At low pressures, the partial entropies of all three species increase with lnP, reflecting the progressive occupation of lattice sites. As pressure continues to rise and the total entropy approaches its maximum, inflection points emerge in the entropy curves of the linear trimers and triangular species. In contrast, the dimers, the smallest species, exhibit a slightly higher local maximum within this region, surpassing the individual contributions of the larger components.

This behavior indicates that dimers contribute most significantly to the total entropy maximum, as they access a greater number of configurations within this lnP range. A comparison between Figure 2 and Figure 4 further reveals that beyond the entropy maximum, the dimers progressively displace the larger species.

A striking feature is observed in the dimer entropy at high total coverage. Although dimers dominate the lattice at elevated pressures, the limited available space severely restricts their configurational degrees of freedom, leading to a sharp decline in the number of accessible states. As a result, beyond the global maximum of the total entropy, their partial entropy decreases and eventually becomes negative as pressure increases. In contrast, the larger species, present in trace amounts, retain configurational freedom by occupying the voids left by the saturated dimers, and their partial entropies increase until reaching a constant positive value.

This behavior can be interpreted as follows: Although present in small quantities, the larger species generate positive entropy by exploiting the voids left by the saturation of dimers. Thus, the total entropy of the ternary mixture remains positive. A similar behavior has been observed in the problem of random mixing of polymer solutions. When the Flory’s approximation is applied [34,37,40], the entropy of the pure polymer becomes negative while the entropy of the mixture is larger than 0 [34,37,40].

It is worth noting that, within the lattice gas framework, the adsorption of *k*-mers on homogeneous surfaces is formally analogous to polymer–solvent binary mixtures: linear *k*-mers correspond to linear polymer chains, while empty lattice sites play the role of monomeric solvent molecules. Accordingly, just as the entropy of a pure polymer becomes negative at low solvent concentrations (i.e., in poor solvents) [34,37], a similar scenario arises in the adsorption of *k*-mers at high surface coverage (i.e., low concentration of empty sites) [40]. In the ternary mixture under study, as discussed above, the entropy associated with dimers becomes negative when the concentrations of the other components in the mixture are very low.

### 3.2. Configurational Entropy per Site for Different Molar Compositions

**Figure 5:**
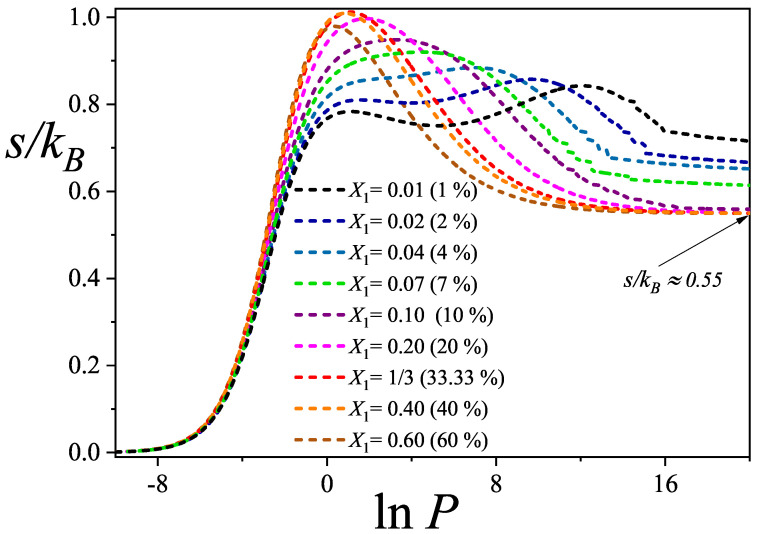
Total configurational entropy per site as a function of lnP for different molar compositions as indicated in the inset. The results correspond to the EA theoretical model developed in Appendix A.

In this section, we examine the behavior of the total configurational entropy per site for varying dimer compositions $X_{1}$, based on the theoretical framework presented in Appendix A (The analysis presented in this section is primarily based on the qualitative behavior of the configurational total entropy of the adsorbed phase as a function of pressure. For this reason, the study is conducted using curves derived from the EA model. Similar results and conclusions could also be obtained from the GD-LC theory). Our objective is to characterize the overall behavior of the mixing entropy in ternary systems. Figure 5 displays the evolution of the total entropy as a function of lnP for dimer compositions ranging from highly dilute systems ($X_{1}=1/100$, i.e., 1 %) to highly concentrated ones ($X_{1}=3/5$, i.e., 60 %).

Each curve in Figure 5 exhibits a characteristic trend: the entropy increases with pressure, reaches a maximum, and subsequently decreases smoothly, eventually approaching a saturation plateau at high pressures. As discussed in Section 3.1, this saturation regime corresponds to a nearly fully occupied lattice, predominantly filled by dimers, with a small residual fraction of trimers. Despite their low coverage, these remaining trimers contribute positively to the entropy due to their configurational freedom. The cumulative effect of the larger species allows the total entropy to remain positive even under conditions of high pressure and coverage. It is worth noting, as previously reported in the literature [53], that the total mixing entropy remains positive in fully occupied lattices, reflecting the residual configurational complexity inherent in densely packed systems.

As shown in Figure 5, for the most dilute compositions ($X_{1}=1\%$ to $X_{1}=4\%$), the total configurational entropy exhibits two distinct maxima across the lnP range. The first maximum occurs at low values of lnP, corresponding to a regime in which the partial coverage of the larger species, namely linear trimers and triangular clusters, increase rapidly, while the dimers accumulate more gradually. The second maximum, discussed in previous paragraphs, emerges at higher pressures and is associated with the gradual replacement of the larger species by dimers. This two-peak structure arises because, at such low dimer concentrations, higher pressures are required to initiate the displacement process.

In the high-pressure regime shown in Figure 5, it is further observed that variations in the saturation values of the total configurational entropy occur only in systems with very low dimer molar fractions. For compositions with $X_{1}\geq 1/10$, the saturation entropy converges to a nearly constant value of approximately $s/k_{B}\approx 0.55$. This trend is consistent with theoretical expectations: at lower dimer concentrations, the residual fraction of larger species, such as trimers and triangular clusters, remains higher, leading to an increase in configurational entropy due to their greater spatial and conformational flexibility on the lattice.

**Figure 6:**
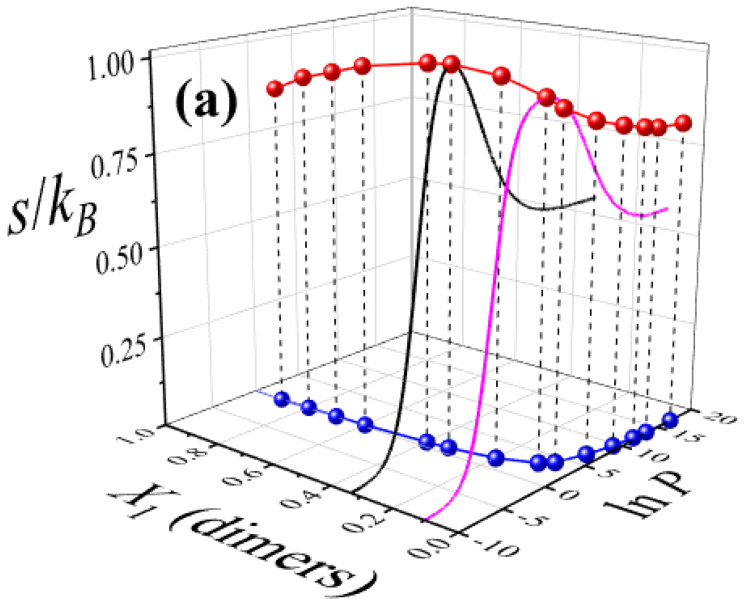

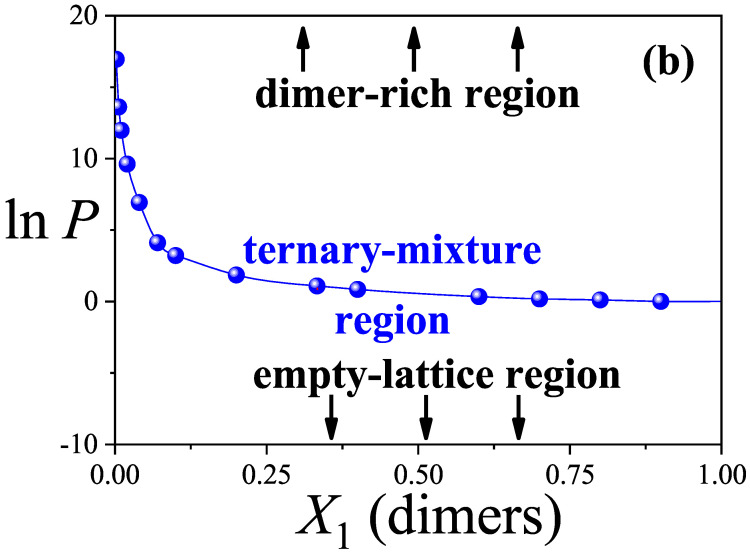
(**a**) The maximum values of the configurational entropy per site (red spheres connected by lines) are shown as a function of the dimer composition $X_{1}$ (in the gas phase) and the corresponding pressure (in ln units) at which these maxima are observed. Black and magenta solid lines correspond to the configurational entropy per site as a function of lnP for $X_{1}=1/3$ and $X_{1}=1/10$, respectively. The black and magenta solid lines represent the configurational entropy per site as a function of lnP for fixed compositions $X_{1}=1/3$ and $X_{1}=1/10$, respectively. These two examples are included for illustration purposes; the remaining entropy profiles are omitted for clarity. (**b**) Blue spheres connected by lines represent the projection of the entropy maxima from part (**a**) onto the ($X_{1}$–lnP) plane. This projection serves as the basis for constructing an entropic phase diagram that delineates the regions dominated by dimers and those characteristic of a ternary mixture (see discussion in the text).

To complete the analysis, it is particularly insightful to examine the maximum values of the mixing entropy. As shown in previous figures, each entropy maximum corresponds to a specific pair of values: lnP and the dimer composition $X_{1}$. This information is summarized in Figure 6a, which presents a phase diagram involving three variables: the configurational entropy per site $s/k_{B}$ (plotted along the *z*-axis), the composition of the smallest species $X_{1}$ (on the *x*-axis), and the logarithm of the pressure lnP (on the *y*-axis) at which the entropy maximum occurs. The maximal values of the configurational entropy are represented by red spheres connected by lines. As illustrative examples, Figure 6a includes the configurational entropy $s/k_{B}$ as a function of lnP for two fixed compositions: $X_{1}=1/3$ (black solid line) and $X_{1}=1/10$ (magenta solid line). The remaining entropy profiles are omitted for clarity. This representation enables clear identification of the different phases that arise in the system. Specifically, (i) a dimer-rich adsorbed phase at high values of lnP, (ii) a ternary mixture phase—comprising dimers, linear trimers, and triangular trimers—at intermediate pressures, and (iii) a nearly empty lattice at low values of lnP.

To better visualize the phase transitions, Figure 6b shows the projection of the entropy maxima onto the $X_{1}$–lnP plane (i.e., the xy-plane), represented by blue spherical markers connected by solid lines. Three distinct regions can be distinguished in this projection. Around the curve of maximum entropy, the system behaves as a ternary mixture, while deviations from this region drive the system either toward a dimer-rich state or toward an almost empty lattice, depending on the trajectory in lnP or $X_{1}$. As previously illustrated in Figure 5, the mixing entropy tends toward a finite constant as lnP increases, corresponding to a nearly saturated lattice of dimers. Conversely, at low lnP, the entropy per site *w* approaches zero, indicating a dilute, nearly empty system.

Finally, it is worth noting that lnP exhibits a nonlinear dependence on the dimer composition $X_{1}$: it decreases steeply at low concentrations ($X_{1}\leq 1/10$) and more gradually at intermediate to high concentrations ($1/4\leq X_{1}\leq 1$). This suggests that, for dilute mixtures, significantly higher pressures are required to drive the system from the ternary mixture region into a dimer-rich adsorbed phase. In contrast, at higher concentrations, relatively small pressure changes suffice to induce this transition.

## 4. Conclusions

In this study, we developed and analyzed a theoretical framework to describe the adsorption of ternary mixtures of polyatomic molecules, specifically dimers, linear trimers, and triangular trimers, on triangular lattices, explicitly accounting for multisite occupancy and excluded volume effects. Our approach combines analytical statistical thermodynamics (EA and GD-LC theoretical models) with grand canonical MC simulations, providing a detailed and consistent description of adsorption phenomena across a wide range of compositions and surface coverages. Several conclusions can be drawn from this study:

- The generalized lattice gas model effectively captures the competitive adsorption behavior driven by molecular size and shape, illustrating the essential role of multisite occupation in realistic surface processes.
- Analytical expressions for thermodynamic quantities, including the Helmholtz free energy, configurational entropy per site, and both total and partial coverages, were derived as functions of pressure within the EA and GD-LC approximations. These theoretical predictions show excellent qualitative agreement with MC simulations. Moreover, the GD-LC theory also shows remarkable quantitative agreement for both adsorption isotherms and configurational entropy, with the corresponding curves from MC simulations and GD-LC being nearly indistinguishable.
- A detailed entropy analysis reveals an entropy-driven displacement mechanism, where dimers progressively replace larger species at higher pressures, maximizing the system’s entropy prior to lattice saturation. In the high-coverage regime, entropy approaches a limiting value dominated by dimer adsorption, in line with previous studies on fully occupied lattices.
- Despite dimers playing a central role in the displacement process, larger molecules contribute cooperatively. Their ability to occupy residual voids left by smaller species supports the preservation of positive entropy and reinforces thermodynamic consistency, particularly in the behavior of mixing entropy.
- The maximum entropy is attained for equimolar compositions, and the entropy landscape enables construction of an“entropic phase diagram” in the composition–pressure–maximum entropy space. This diagram delineates regions where competitive displacement is either enhanced or suppressed, offering a predictive tool for controlling surface composition and illustrating the richness of configurational possibilities in such systems.

Overall, this study provides a comprehensive understanding of the role of entropy in multicomponent adsorption systems with multisite occupancy. The theoretical framework presented here not only shows excellent agreement with simulation data but also offers predictive capabilities for more complex scenarios. Future extensions could incorporate lateral interactions, surface heterogeneity, or more intricate adsorbate shapes and lattice geometries, thereby expanding the applicability of this approach to technologically relevant adsorption systems. Furthermore, we aim to move toward the application of more advanced theoretical tools to address the challenges posed by polyatomic mixtures. Promising avenues include the recently developed Multiple Exclusion statistics framework [48,49,50], as well as extensions of Lattice Fundamental Measure Theory that explicitly account for triangular trimers and go beyond the conventional “0D” approximation [54,55,56,57,58,59,62].

## Data Availability

Dataset available on request from the authors.

## Appendix A. Thermodynamic Functions of Multicomponent Lattice Gases with Hard-Core Interactions

Consider a one-dimensional lattice composed of *M* sites, each separated by a constant distance *a*, under periodic boundary conditions. We assume M→∞. As mentioned at the beginning of Section 2, a *j*-component mixture is considered. The system consists of $N_{1}$ particles of species 1, $N_{2}$ of species 2, and so on, up to $N_{j}$ of species *j*. Each particle of type *i* is modeled as a linear chain, or $k_{i}$-mer, comprising $k_{i}$ identical units, where each unit occupies a single site on the lattice. As a result, one $k_{i}$-mer spans $k_{i}$ adjacent lattice sites when adsorbed. The only interaction considered is hard-core exclusion, meaning that no two $k_{i}$-mers may overlap or occupy the same site. Under these assumptions, the canonical partition function $Q(M,N_{1},N_{2},\ldots ,N_{j},T)$ is expressed as follows:

(A1) $$Q\left(M,N_{1},N_{2},\ldots ,N_{j},T\right)=\Omega \left(M,N_{1},N_{2},\ldots ,N_{j}\right)\exp\left[-\frac{E(M,N_{1},N_{2},\ldots ,N_{j})}{k_{B}T}\right],$$

where $\Omega (M,N_{1},N_{2},\ldots ,N_{j})$ represents the total number of spatial arrangements consistent with the imposed constraints, $E(N_{1},N_{2},\ldots ,N_{j})$ denotes the total energy of interaction between the adsorbate molecules and the substrate, *T* is the absolute temperature, and $k_{B}$ is Boltzmann’s constant.

The term $\Omega (M,N_{1},N_{2},\ldots ,N_{j})$ can be derived as the total number of permutations of the $N_{1}$ indistinguishable $k_{1}$-mers, $N_{2}$ indistinguishable $k_{2}$-mers, …, and $N_{j}$ indistinguishable $k_{j}$-mers out of $n_{e}$ entities, where $n_{e}$ is given by

(A2) $$\begin{array}{ccc} n_{e} & = & N_{1}+N_{2}+\ldots +N_{j}+N_{0}\\ & = & \sum_{i=1}^{j}N_{i}+M-\sum_{i=1}^{j}k_{i}N_{i}=M-\sum_{i=1}^{j}\left(k_{i}-1\right)N_{i}, \end{array}$$

with $N_{0}$ denoting the number of vacant sites. Accordingly,

(A3) $$\Omega \left(M,N_{1},N_{2},\ldots ,N_{j}\right)=\left(\begin{array}{c} n_{e}\\ N \end{array}\right)=\frac{\left[M-\sum_{i=1}^{j}\left(k_{i}-1\right)N_{i}\right]!}{N_{1}!N_{2}!\ldots N_{j}!\left[M-\sum_{i=1}^{j}k_{i}N_{i}\right]!}.$$

Let us now generalize this to a lattice of higher dimensionality characterized by a coordination number *c* (e.g., c=3 for a honeycomb structure, c=4 for a square lattice, and c=6 for a triangular one). In this case, the configurational contribution, denoted as $\Omega_{c}\left(M,N_{1},N_{2},\ldots ,N_{j}\right)$, is approximated by considering a random, uncorrelated distribution of adsorbed species. Following methodologies proposed in prior studies [34,35,37,40,60,61], this multidimensional configurational factor is related to the one-dimensional result via

(A4) $$\Omega_{c}\left(M,N_{1},N_{2},\ldots ,N_{j}\right)\approx {\left[K_{1}\left(c,k_{1}\right)\right]}^{N_{1}}{\left[K_{2}\left(c,k_{2}\right)\right]}^{N_{2}}\ldots {\left[K_{j}\left(c,k_{j}\right)\right]}^{N_{j}}\Omega_{c=2}\left(M,N_{1},N_{2},\ldots ,N_{j}\right)$$

where $K_{i}\left(c,k_{i}\right)$ corresponds to the number of configurations available per lattice site to a $k_{i}$-mer at zero surface coverage. The approximation presented in Equation (A4) is known as the Extension Ansatz (EA) approximation [47]. This quantity generally depends on both the connectivity of the lattice and the geometric characteristics of the adsorbed molecule. For straight, rigid $k_{i}$-mers (linear particles with $k_{i}$ monomeric units), the available orientations reduce to $K_{i}\left(c,k_{i}\right)=c/2$ (this expression is only valid for $k_{i}\geq 2$; for monomer adsorption $K_{i}\left(c,k_{i}=1\right)=1$.

On the other hand, $E(M,N_{1},N_{2},\ldots ,N_{j})$ can be written as

(A5) $$E\left(M,N_{1},N_{2},\ldots ,N_{j}\right)=\sum_{i=1}^{j}\epsilon_{i}N_{i},$$

where $\epsilon_{i}$ denotes the binding energy between a $k_{i}$-mer and the surface.

The Helmholtz free energy $F(M,N_{1},N_{2},\ldots ,N_{j},T)$ is related to $Q(M,N_{1},N_{2},\ldots ,N_{j},T)$ through

(A6) $$\begin{array}{ccc} \beta F(M,N_{1},N_{2},\ldots ,N_{j},T) & = & -\ln Q(M,N_{1},N_{2},\ldots ,N_{j},T)\\ & = & -\ln\Omega_{c}\left(M,N_{1},N_{2},\ldots ,N_{j}\right)+\beta \sum_{i=1}^{j}\epsilon_{i}N_{i}, \end{array}$$

where $\beta ={\left(k_{B}T\right)}^{-1}$. From Equations (A3), (A4), and (A6),

(A7) $$\begin{array}{ccc} \beta F(M,N_{1},N_{2},\ldots ,N_{j},T) & = & -\sum_{i=1}^{j}N_{i}\ln\left[K_{i}\left(c,k_{i}\right)\right]-\ln\left[M-\sum_{i=1}^{j}\left(k_{i}-1\right)N_{i}\right]!\\ & & +\sum_{i=1}^{j}\left[\ln\left(N_{i}\right)!\right]+\ln\left[M-\sum_{i=1}^{j}k_{i}N_{i}\right]!+\beta \sum_{i=1}^{j}\epsilon_{i}N_{i}. \end{array}$$

Using the Stirling approximation

(A8) $$\begin{array}{ccc} \beta F(M,N_{1},N_{2},\ldots ,N_{j},T) & = & -\sum_{i=1}^{j}N_{i}\ln\left[K_{i}\left(c,k_{i}\right)\right]\\ & & -\left[M-\sum_{i=1}^{j}\left(k_{i}-1\right)N_{i}\right]\ln\left[M-\sum_{i=1}^{j}\left(k_{i}-1\right)N_{i}\right]\\ & & +\sum_{i=1}^{j}\left[N_{i}\ln\left(N_{i}\right)\right]+\left[M-\sum_{i=1}^{j}k_{i}N_{i}\right]\ln\left[M-\sum_{i=1}^{j}k_{i}N_{i}\right]\\ & & +\beta \sum_{i=1}^{j}\epsilon_{i}N_{i}. \end{array}$$

The configurational entropy *S*, and the chemical potential corresponding to the *i*-species $\mu_{i,ads}$, can be calculated as [34]

(A9) $$S\left(M,N_{1},N_{2},\ldots ,N_{j},T\right)=-{\left(\frac{\partial F}{\partial T}\right)}_{M,N_{j}^{\prime }s},$$

and

(A10) $$\mu_{i,ads}={\left(\frac{\partial F}{\partial N_{i}}\right)}_{T,M,N_{j}^{\prime }s\;\left(j\neq i\right)}\;\;\;\;\;\;\;\;\left(i=1,2,\ldots ,j\right).$$

From Equations (A8)–(A10) it follows that

(A11) $$\begin{array}{ccc} \frac{S(M,N_{1},N_{2},\ldots ,N_{j},T)}{k_{B}} & = & +\sum_{i=1}^{j}N_{i}\ln\left[K_{i}\left(c,k_{i}\right)\right]\\ & & +\left[M-\sum_{i=1}^{j}\left(k_{i}-1\right)N_{i}\right]\ln\left[M-\sum_{i=1}^{j}\left(k_{i}-1\right)N_{i}\right]\\ & & -\sum_{i=1}^{j}\left[N_{i}\ln\left(N_{i}\right)\right]-\left[M-\sum_{i=1}^{j}k_{i}N_{i}\right]\ln\left[M-\sum_{i=1}^{j}k_{i}N_{i}\right], \end{array}$$

and

(A12) $$\begin{array}{ccc} \beta \left(\mu_{i,ads}-\epsilon_{i}\right) & = & -\ln\left[K_{i}\left(c,k_{i}\right)\right]+\left(k_{i}-1\right)\ln\left[M-\sum_{l=1}^{j}\left(k_{l}-1\right)N_{l}\right]\\ & & +\ln\left(N_{i}\right)-k_{i}\ln\left[M-\sum_{l=1}^{j}k_{l}N_{l}\right]\;\;\;\;\;\;\;\;\left(i=1,2,\ldots ,j\right). \end{array}$$

Then, by defining the partial coverage of the species *i*, $\theta_{i}=k_{i}N_{i}/M$, the free energy per site f=F/M, and the configurational entropy per site s=S/M, Equations (A8), (A11), and (A12) can be rewritten in terms of the intensive variables $\theta_{1}$, $\theta_{2}$, …, $\theta_{j}$, and *T*,

(A13) $$\begin{array}{ccc} \beta f(\theta_{1},\theta_{2},\ldots ,\theta_{j},T) & = & -\sum_{i=1}^{j}\frac{\theta_{i}}{k_{i}}\ln\left[K_{i}\left(c,k_{i}\right)\right]\\ & & -\left[1-\sum_{i=1}^{j}\left(\frac{k_{i}-1}{k_{i}}\right)\theta_{i}\right]\ln\left[1-\sum_{i=1}^{j}\left(\frac{k_{i}-1}{k_{i}}\right)\theta_{i}\right]\\ & & +\sum_{i=1}^{j}\left[\frac{\theta_{i}}{k_{i}}\ln\left(\frac{\theta_{i}}{k_{i}}\right)\right]+\left(1-\sum_{i=1}^{j}\theta_{i}\right)\ln\left(1-\sum_{i=1}^{j}\theta_{i}\right)\\ & & +\beta \sum_{i=1}^{j}\epsilon_{i}\frac{\theta_{i}}{k_{i}}, \end{array}$$

(A14) $$\begin{array}{ccc} \frac{s(\theta_{1},\theta_{2},\ldots ,\theta_{j},T)}{k_{B}} & = & +\sum_{i=1}^{j}\frac{\theta_{i}}{k_{i}}\ln\left[K_{i}\left(c,k_{i}\right)\right]\\ & & +\left[1-\sum_{i=1}^{j}\left(\frac{k_{i}-1}{k_{i}}\right)\theta_{i}\right]\ln\left[1-\sum_{i=1}^{j}\left(\frac{k_{i}-1}{k_{i}}\right)\theta_{i}\right]\\ & & -\sum_{i=1}^{j}\left[\frac{\theta_{i}}{k_{i}}\ln\left(\frac{\theta_{i}}{k_{i}}\right)\right]-\left(1-\sum_{i=1}^{j}\theta_{i}\right)\ln\left(1-\sum_{i=1}^{j}\theta_{i}\right), \end{array}$$

and

(A15) $$\begin{array}{ccc} \beta \left(\mu_{i,ads}-\epsilon_{i}\right) & = & -\ln\left[K_{i}\left(c,k_{i}\right)\right]+\left(k_{i}-1\right)\ln\left[1-\sum_{l=1}^{j}\left(\frac{k_{l}-1}{k_{l}}\right)\theta_{l}\right]\\ & & +\ln\left(\frac{\theta_{i}}{k_{i}}\right)-k_{i}\ln\left[1-\sum_{l=1}^{j}\theta_{l}\right]\;\;\;\;\;\;\;\;\left(i=1,2,\ldots ,j\right). \end{array}$$

At equilibrium, the chemical potential of the adsorbed and gas phase are equal. Then,

(A16) $$\mu_{i,ads}=\mu_{i,gas}\;\;\;\;\;\;\;\;\left(i=1,2,\ldots ,j\right),$$

where $\mu_{i,gas}$ corresponds to $k_{i}$-mers in the gas phase.

The chemical potential of each kind of molecule in an ideal gas mixture, at temperature *T* and pressure *P*, is

(A17) $$\beta \mu_{i,gas}=\beta \mu_{i}^{0}+\ln X_{i}P\;\;\;\;\;\;\;\;\left(i=1,2,\ldots ,j\right),$$

where $\mu_{i}^{0}$ and $X_{i}$ are the standard chemical potential and the mole fraction of $k_{i}$-mers, respectively. In addition, $\sum_{i=1}^{j}X_{i}=1$.

From Equations (A15), (A16), and (A17), the partial adsorption isotherms can be obtained,

(A18) $$\begin{array}{c} -\ln\left[K_{i}\left(c,k_{i}\right)\right]+\left(k_{i}-1\right)\ln\left[1-\sum_{l=1}^{j}\left(\frac{k_{l}-1}{k_{l}}\right)\theta_{l}\right]+\ln\left(\frac{\theta_{i}}{k_{i}}\right)\\ -k_{i}\ln\left[1-\sum_{l=1}^{j}\theta_{l}\right]+\beta \left(\epsilon_{i}-\mu_{i}^{0}\right)-\ln X_{i}-\ln P=0\;\;\;\;\;\;\;\;\left(i=1,2,\ldots ,j\right). \end{array}$$

## Appendix B. Ideal Adsorbed Solution Theory (IAST)

IAST is based on the assumption that the adsorbed phase can be treated as an ideal solution of the adsorbed components. The fundamental equation of IAST theory is the analog of Raoult’s law for the vapor–liquid equilibrium:

(A19) $$Py_{i}=P_{i}^{0}\left(\pi \right)x_{i},$$

where *P* is the total pressure and $P_{i}^{0}\left(\pi \right)$ is the sorption pressure of each pure component *i*, which generates the same spreading pressure π as the mixture. $y_{i}$ and $x_{i}$ are the molar fractions of component *i* in the fluid phase and the adsorbed phase, respectively. Thus,

(A20) $$x_{i}=\frac{\theta_{i}}{\sum_{j}\theta_{j}},$$

where the sum extends over the number of components in the mixture. For the ternary mixture analyzed in the main text, we have

(A21) $$x_{1(2)[3]}=\frac{\theta_{1(2)[3]}}{\theta_{1}+\theta_{2}+\theta_{3}},\;\;\;\;\left[1\;(\mathrm{dimer}),\;2\;(\mathrm{linear}\;\mathrm{trimer})\;\mathrm{and}\;3\;(\mathrm{triangular}\;\mathrm{trimer})\right].$$

The spreading pressure is defined by the Gibbs adsorption isotherm [92]:

(A22) $$\frac{\pi }{k_{B}T}=\int_{0}^{P_{i}^{0}\left(\pi \right)}\frac{\theta_{i}\left(P\right)}{P}\;dP,$$

where $\theta_{i}\left(P\right)$ is the isotherm of each pure component. Thus, the total isotherm obtained can be written as follows:

(A23) $$\theta =\sum_{j}\theta_{j}=\frac{1}{\sum_{i}x_{i}/\theta_{i}\left(P_{i}^{0}\right)}.$$

Finally, by using Equation (A23) together with the pure-component isotherms derived from Equation (A15) (and compiled in Table A1), it is possible to compute the total adsorption isotherm of the mixture using IAST theory.

**Table A1:** Adsorption isotherms for pure species described by Equation (A15).

*i*	Species	$k_{i}$	$K_{i}\left(c,k_{i}\right)$	Adsorption Isotherm Equation
1	dimer	2	3	$\beta \mu_{1}=-\ln3+\ln\left(1-\frac{1}{2}\theta \right)+\frac{\theta }{2}-2\ln\left(1-\theta \right)$
2	linear trimer	3	3	$\beta \mu_{2}=-\ln3+\ln\left(1-\frac{2}{3}\theta \right)+\frac{\theta }{3}-3\ln\left(1-\theta \right)$
3	triangular trimer	3	2	$\beta \mu_{3}=-\ln2+\ln\left(1-\frac{2}{3}\theta \right)+\frac{\theta }{3}-3\ln\left(1-\theta \right)$
